# Supplementary figures and images for: The complete mitochondrial genomes of five longicorn beetles (Coleoptera: Cerambycidae) and phylogenetic relationships within Cerambycidae
Source: PeerJ. 2019 Sep 5;7:e7633. doi: 10.7717/peerj.7633 (PMC6732212; doi:10.7717/peerj.7633)

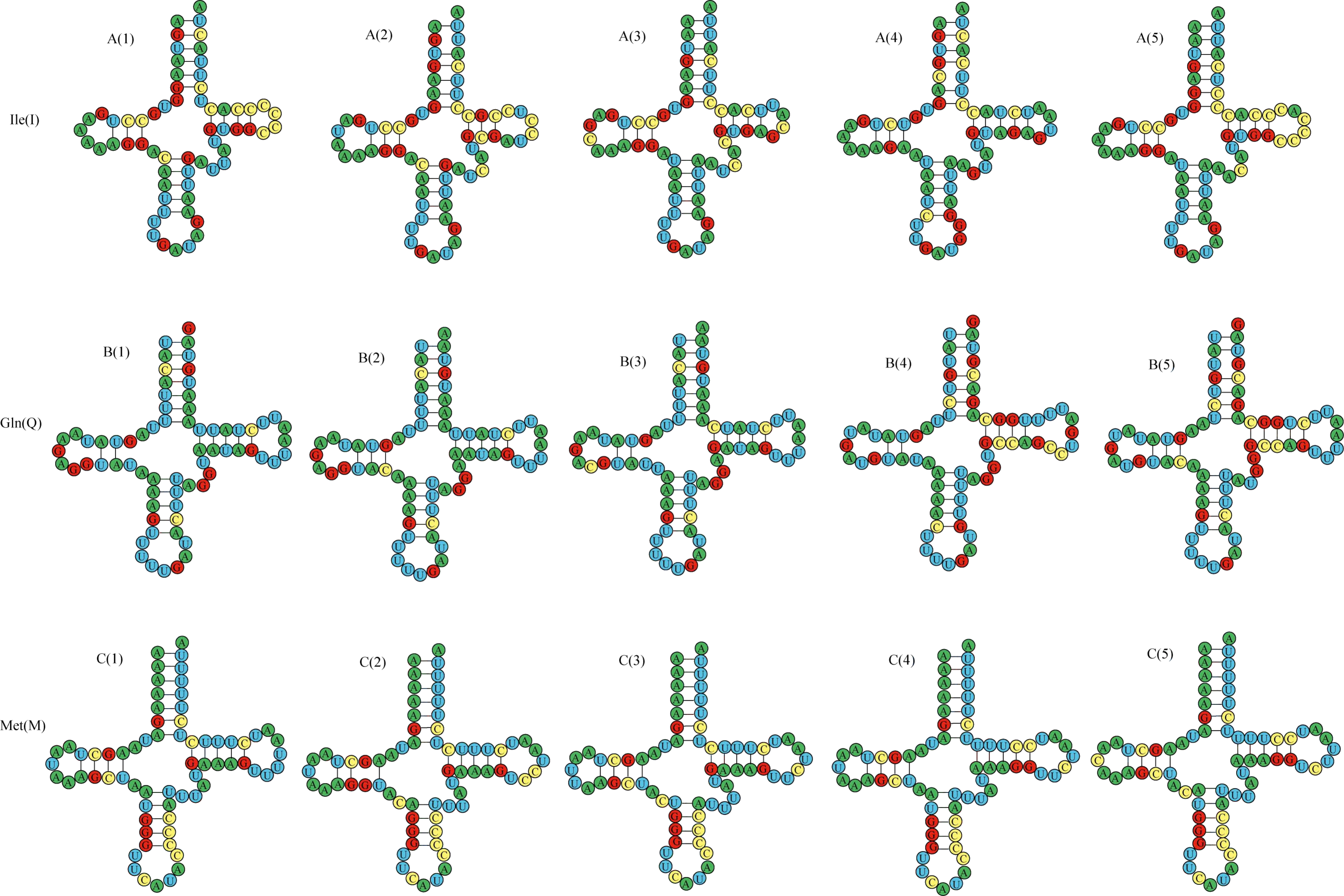

$$\text{Tyr}(\mathbf{Y})$$
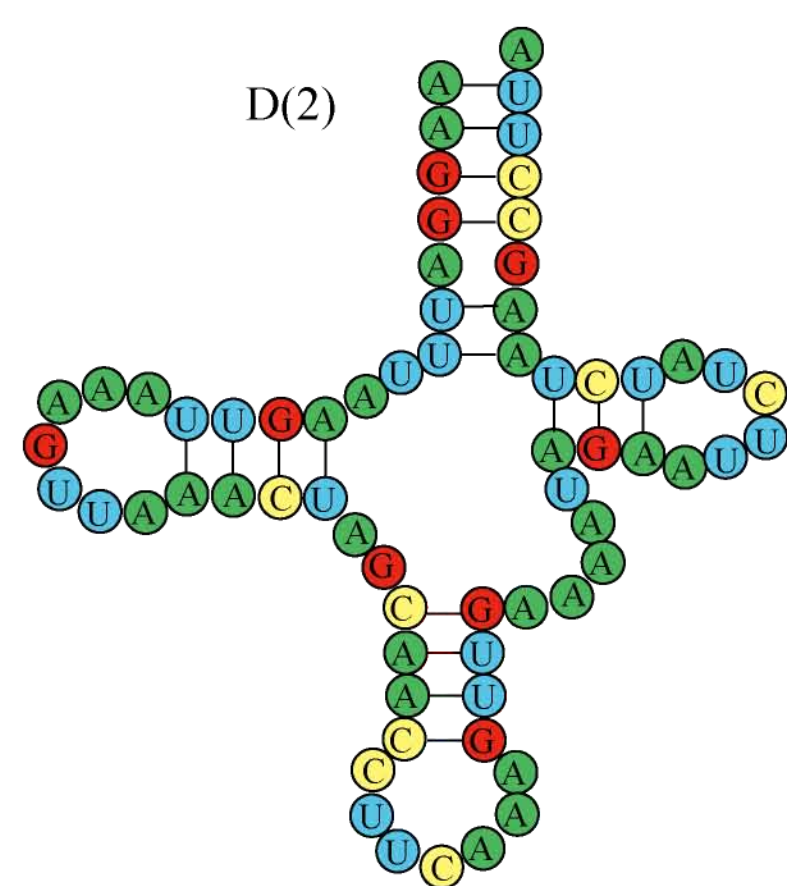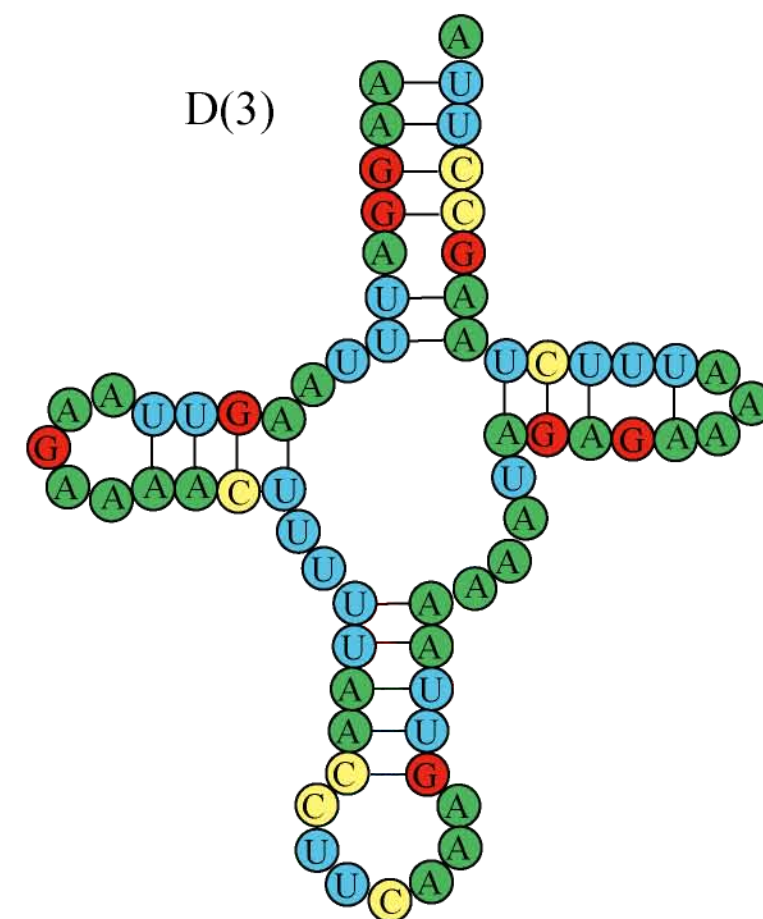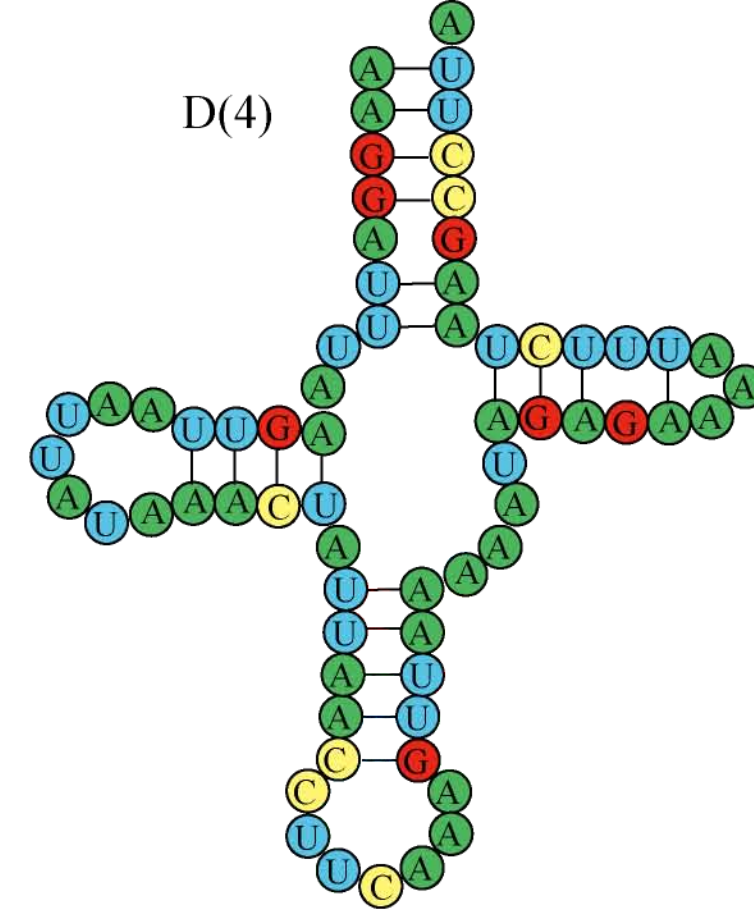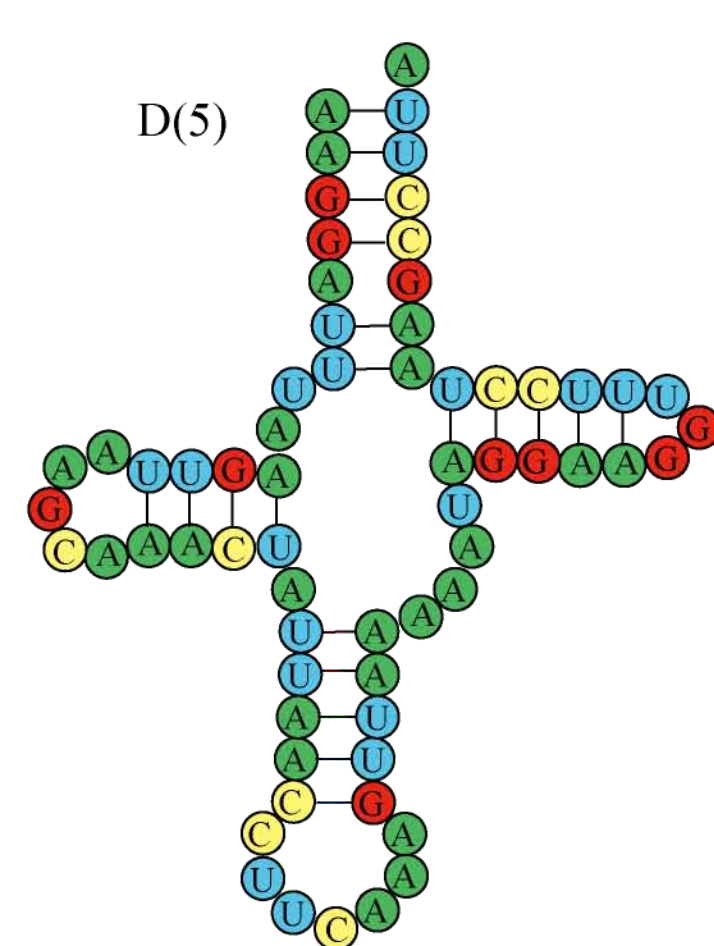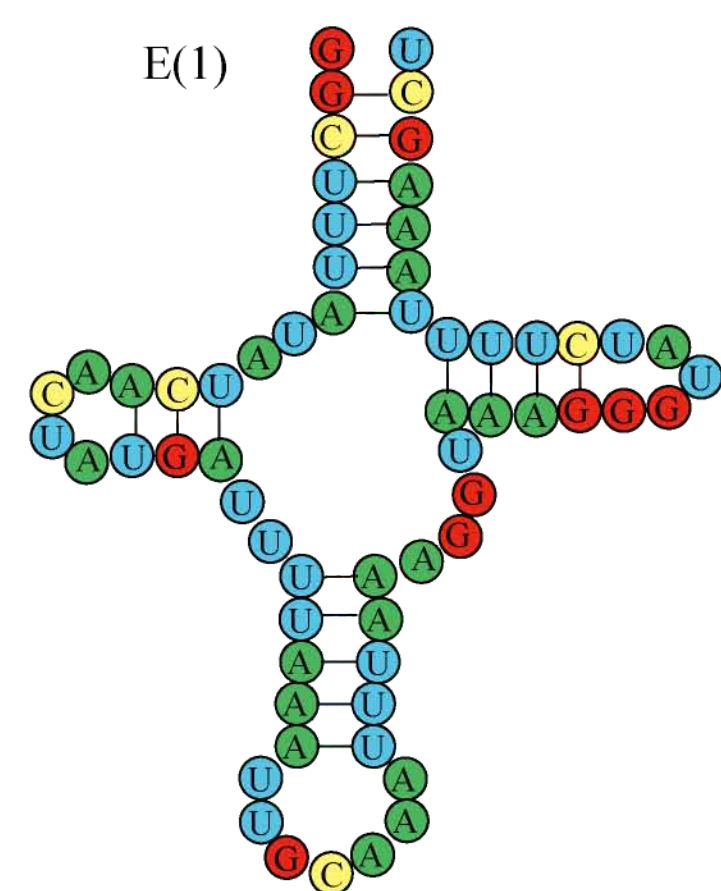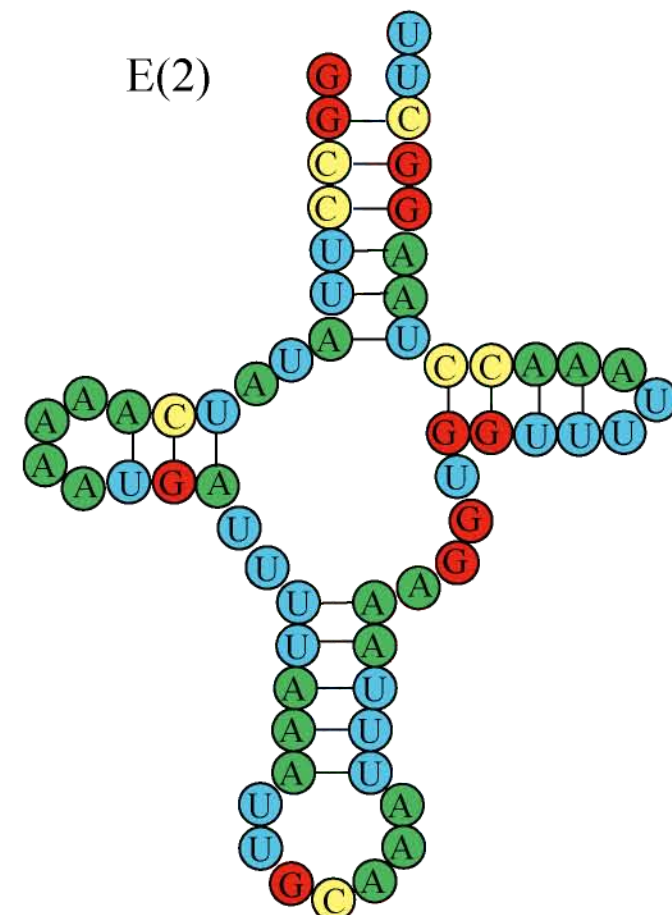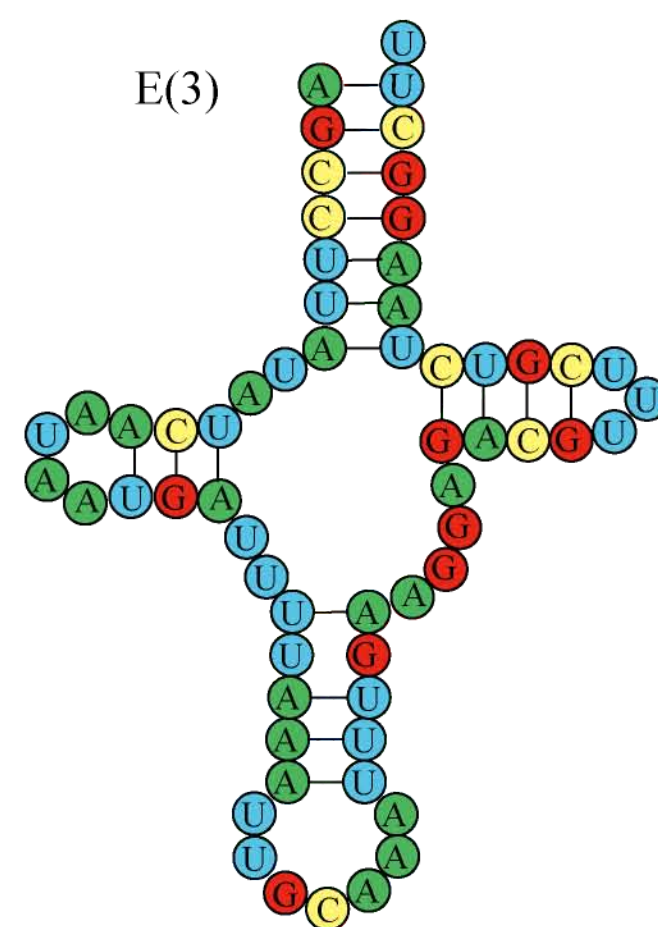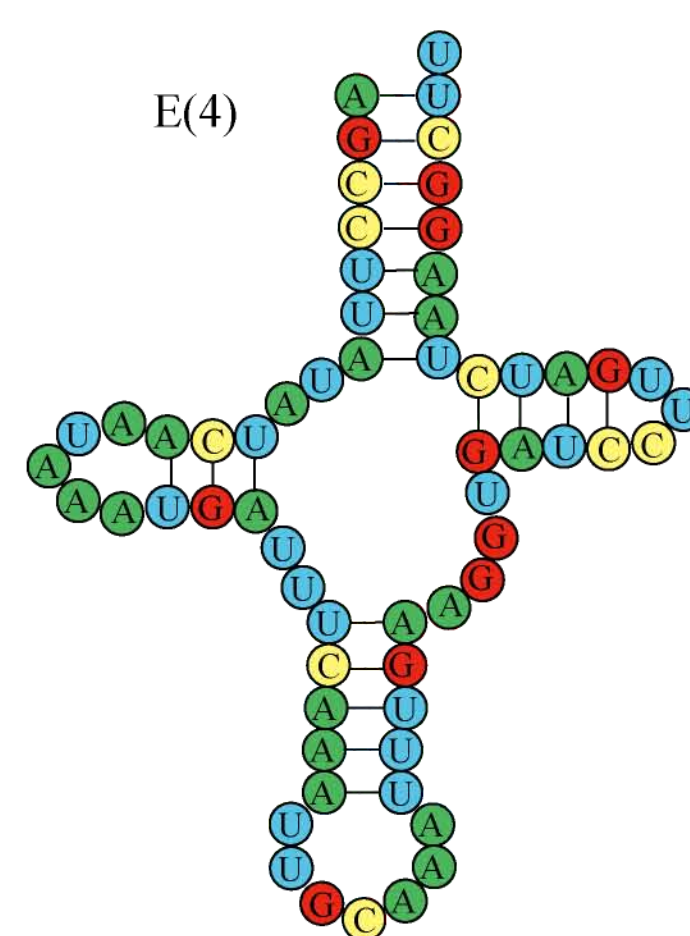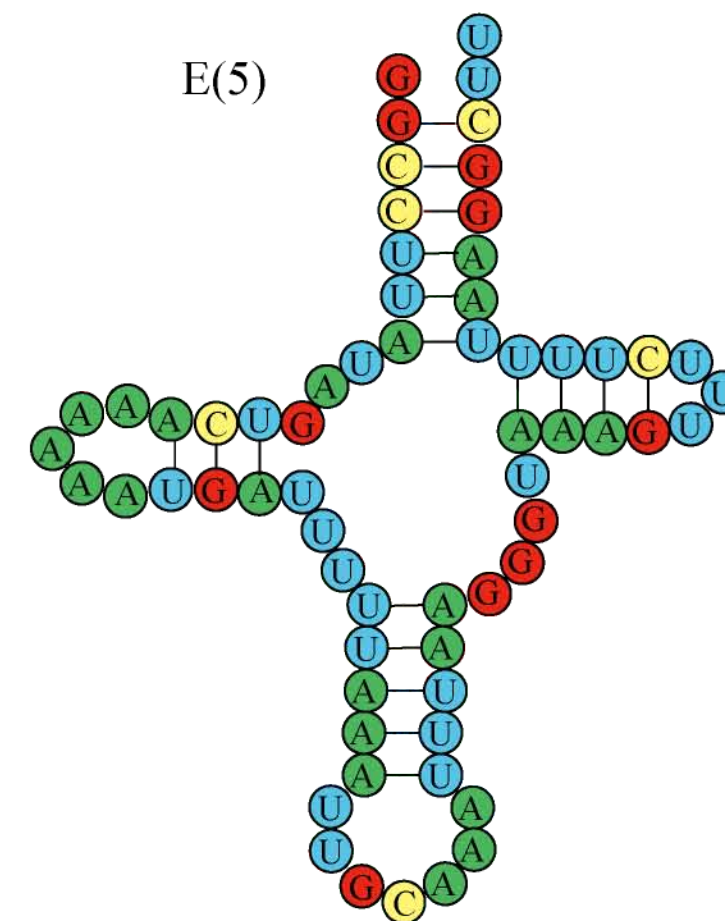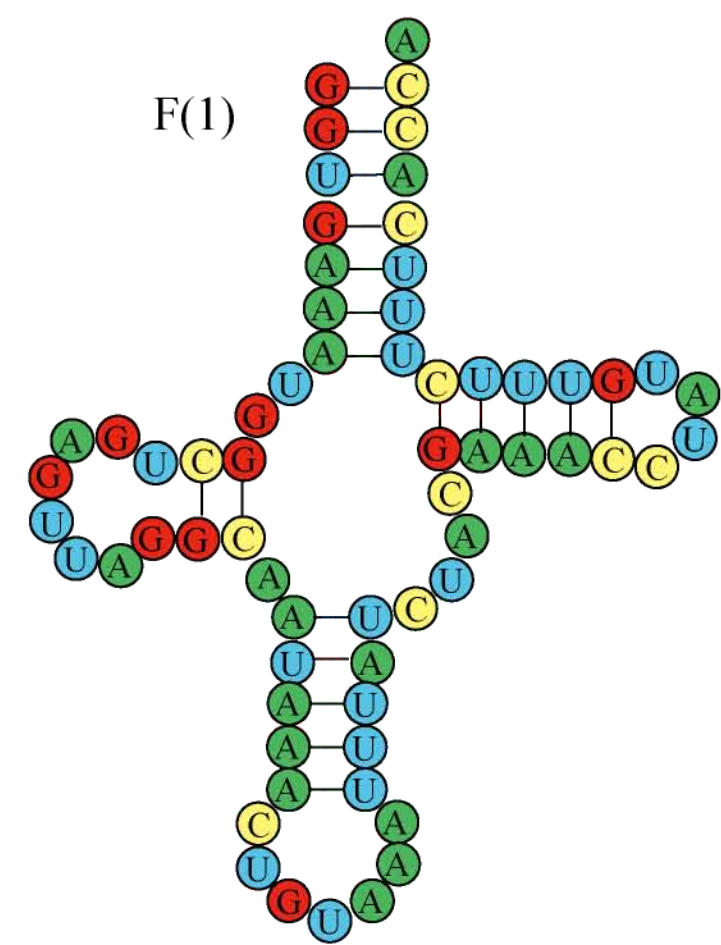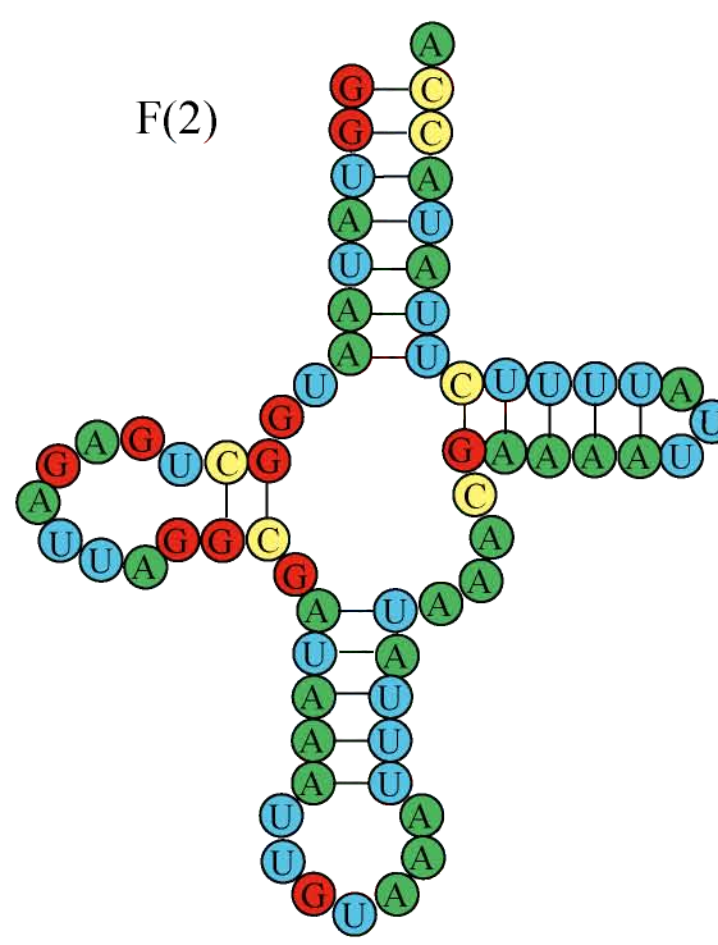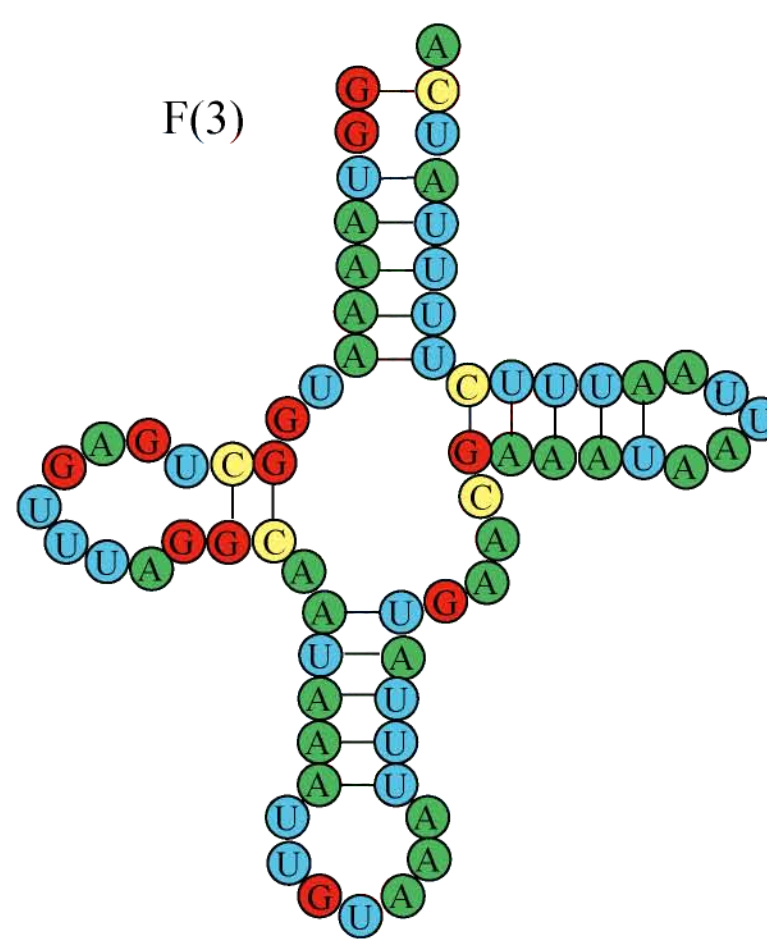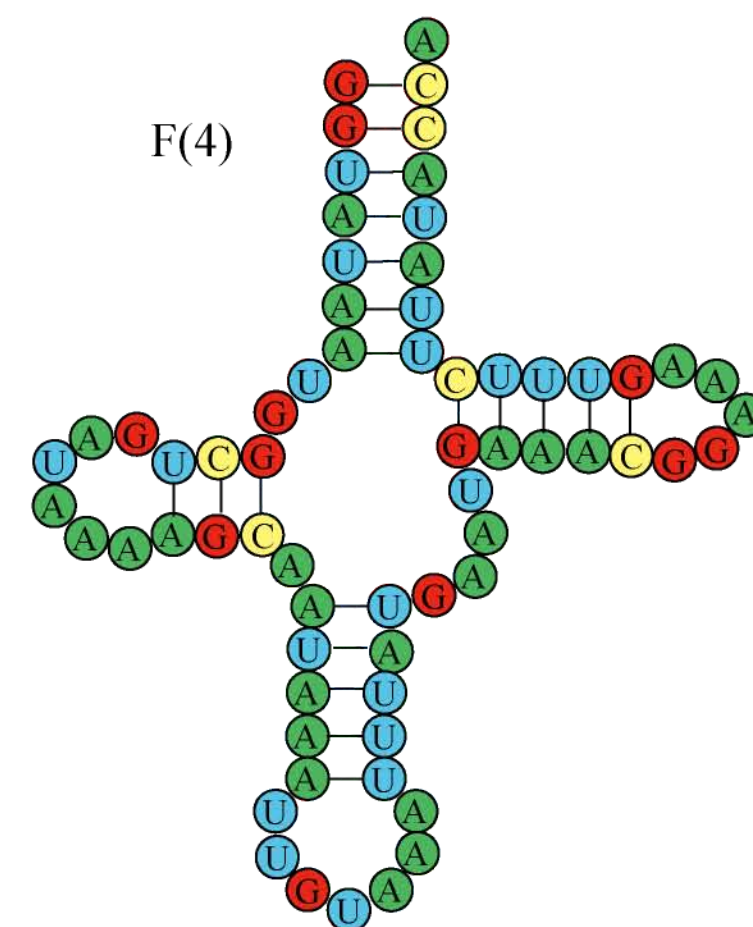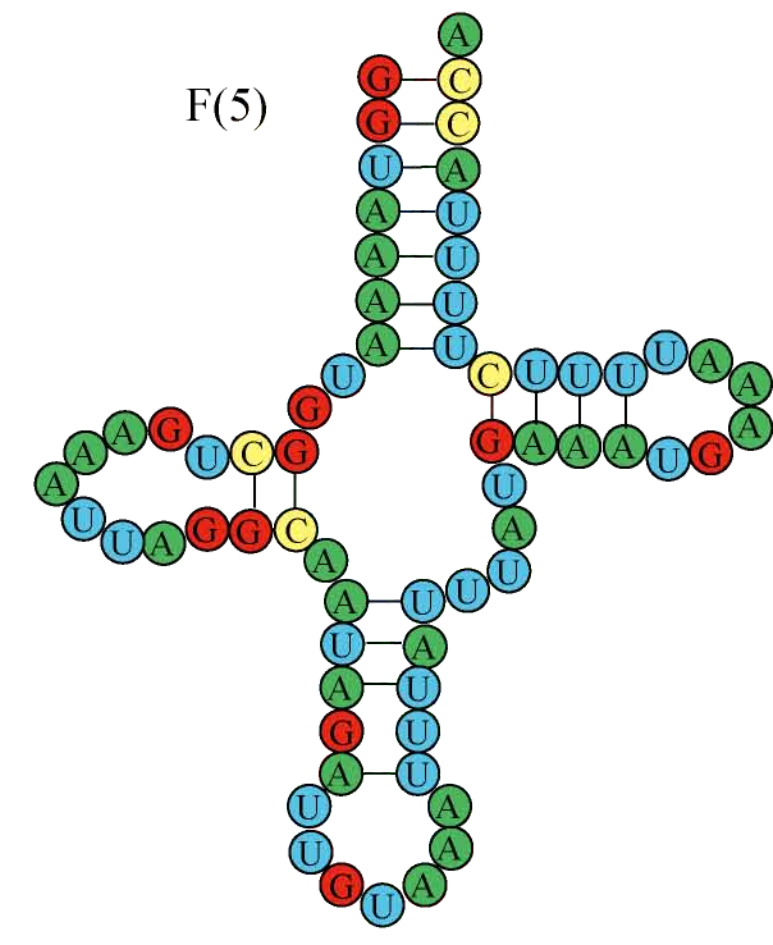

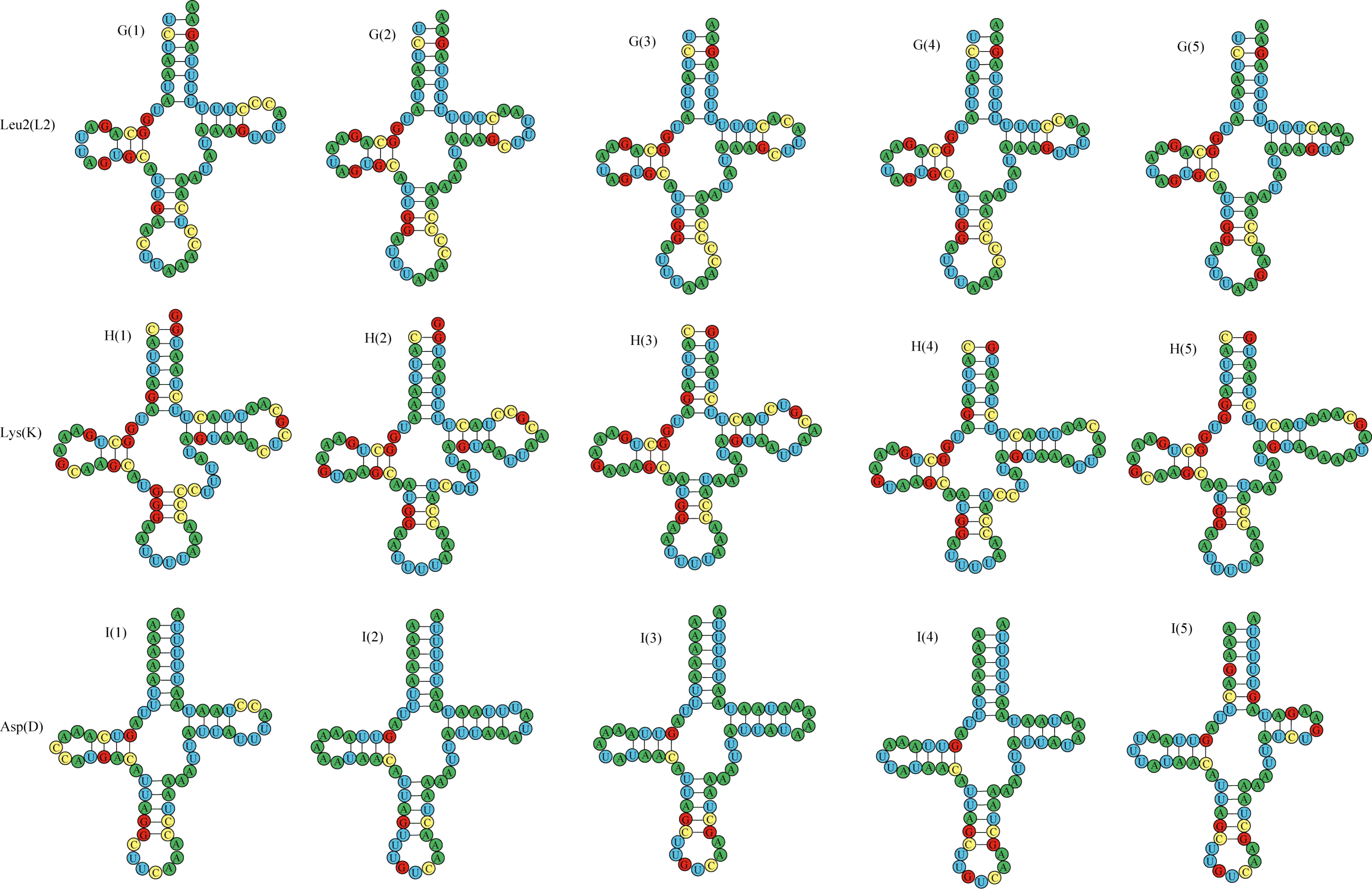

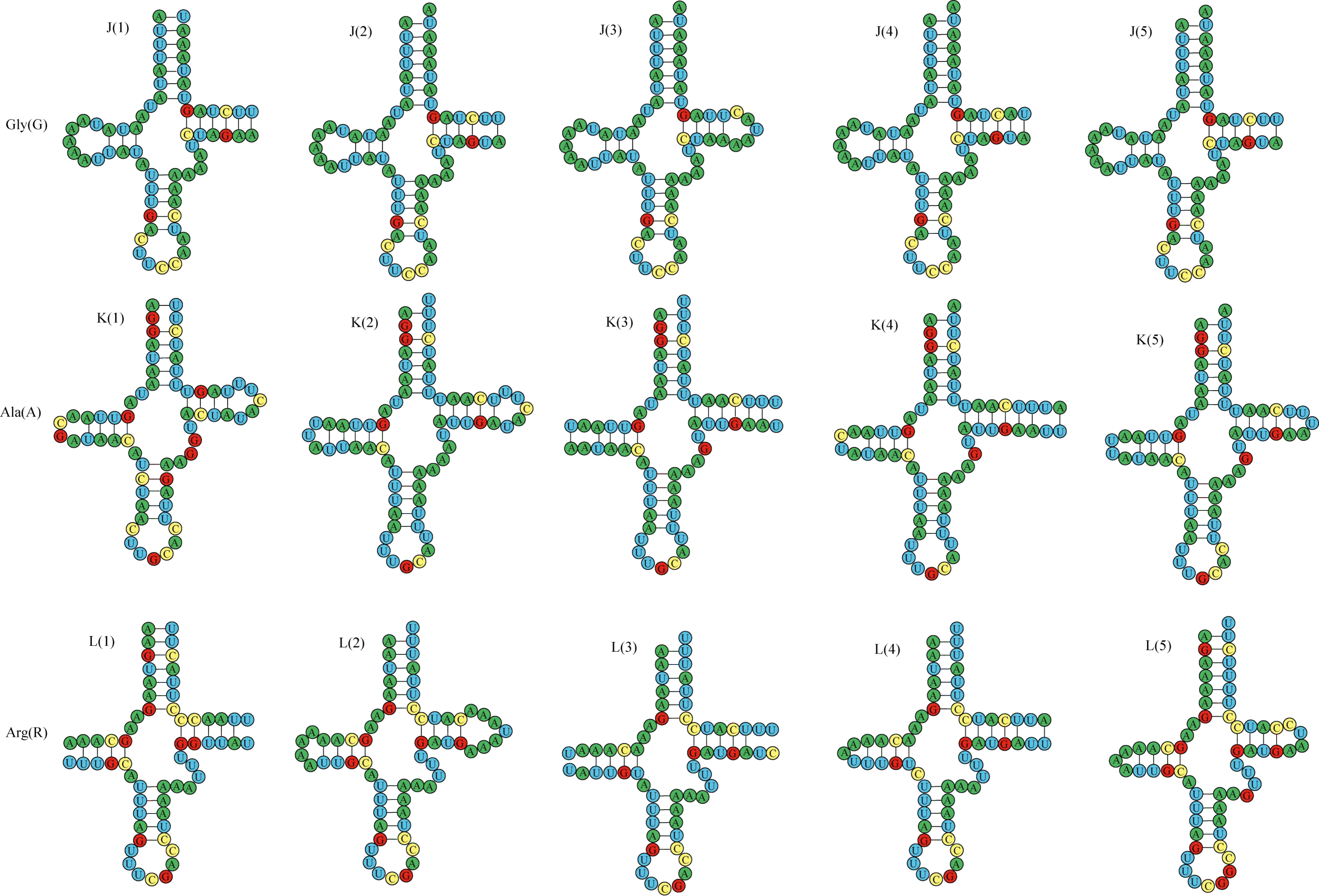

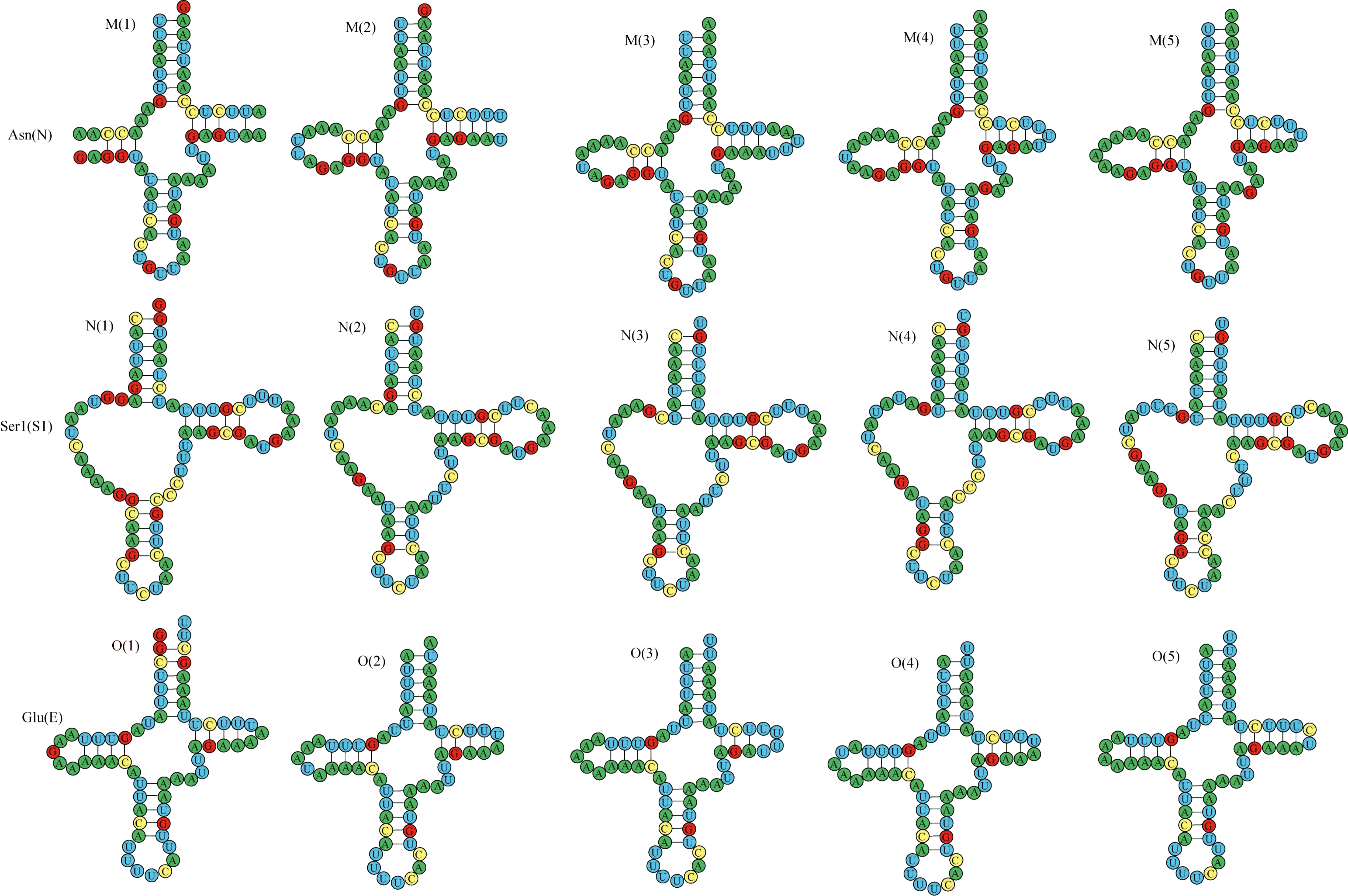

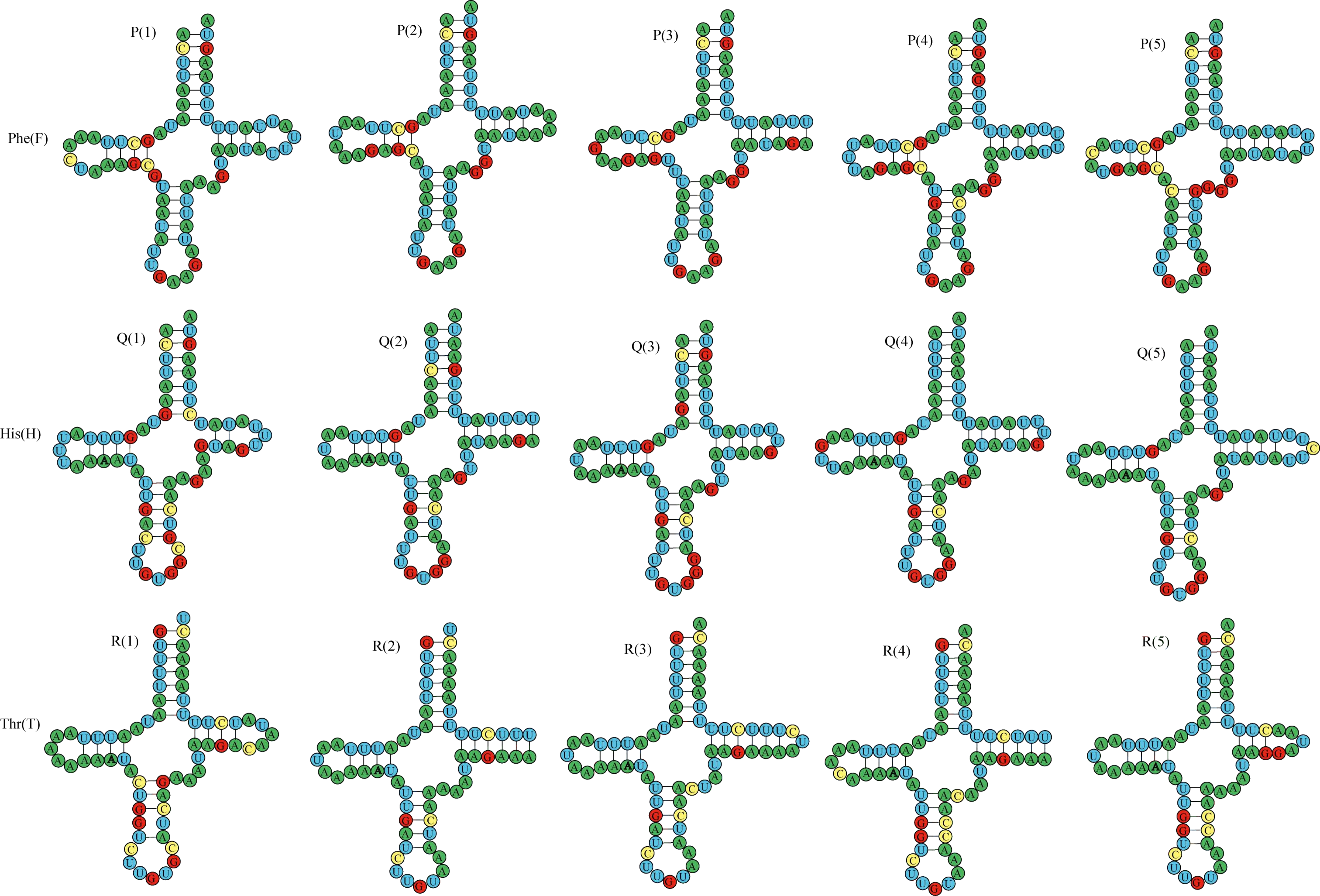

Supplement: Figure S6 — (1): O. yaoshana (2): T. croccocincta (3): B. succinctor (4): N. carinicollis (5): Pterolophia sp. ZJY-2019 A: trnI ; B: trnQ; C: trnM; D: trnW; E: trnC; F: trnY; G: trnL (UUA); H: trnK; I: trnD; J: trnG; K: trnA; L: trnR; M: trnN; N: trnS (AGN); O: trnE; P: trnF; Q: trnH; R: trnT; S: trnP; T: trnS (UCN); U: trn L (CUA); V: trnV. [file peerj-07-7633-s007.pdf]
