# Supplementary material for: The complete mitochondrial genomes of five longicorn beetles (Coleoptera: Cerambycidae) and phylogenetic relationships within Cerambycidae
Source: PeerJ. 2019 Sep 5;7:e7633. doi: 10.7717/peerj.7633 (PMC6732212; doi:10.7717/peerj.7633)
Supplement: Supplemental Information 2 [file peerj-07-7633-s008.docx]

| Primers | Number | Primer name | Sequence (5’-3’) | References |
| --- | --- | --- | --- | --- |
| Universal primers | 1 | FY-J-214 | AAGCTHDTRGGTTCATAYCCY | Simon, 2006 |
|  |  | FY-N-1873 | AANGGDGGRTAVACNGTYCA | Simon, 2006 |
|  | 2 | FY-J-1423 | ACDAAYCAYAARGAYATYGG | Simon, 1994  et al., 1994 |
|  |  | FY-N-2329 | ACDGTRAAYATRTGRTGNGCYCA | Simon, 1994 |
|  | 3 | FY-J-2198 | TATTHTGATTYTTYGGNCAYCCHGAAGT | Simon, 2006 |
|  |  | FY-N-3705 | GCYCCRCARATTTCNGAACATTG | Simon, 2006 |
|  | 4 | FY-J-4463 | TTYGCHCAYYTDGTNCCNCARGG | Simon, 2006 |
|  |  | FY-N-5748 | GGRTCRAANCCRCAYTCRAANGG | Simon, 2006 |
|  | 5 | TN-J-6172 | AGAGGTATGTCACTGTTAATGA | Simon, 2006 |
|  |  | TN-N-7211 | TTAAAGCTTTAYTATTTATRTGYGC | Simon, 2006 |
|  | 6 | FY-J-7572 | AAANGGRATYTGNGCDCTYTTHGT | Simon, 2006 |
|  |  | FY-N-8741 | AYTTCRATNGYTTGHCCHT | Simon, 2006 |
|  | 7 | N4-J-9172 | CGTTCAGGYTGRTAGCCYCA | Simon, 2006 |
|  |  | N4-N-10608 | CCAAGTARAGAWCCAAARTTTCA | Simon, 2006 |
|  | 8 | FY-J-10885 | AYGTYCTRCCYTGRGGWCARATRTC | Simon, 1994 |
|  |  | FY-N-12964 | TTACCTTARGGATAACAGCRTAW | Zhang et al., 2018 |
|  | 9 | FY-J-12831 | CGGTYTGAACTCAGATCATGTA | Simon, 1994 |
|  |  | FY-N-13889 | KTACCTTKTGTATCAGGGTT | Simon, 2006 |
|  | 10 | LR-J13900 | TTTGATTAACYCTGATACAMAAG | Simon, 1994 |
|  |  | LR-N14745 | GTGCCAGCAGYYGCGGTTANAC | Simon, 2006 |
|  | 11 | J-14610 | ATAATAGGGTATCTAATCCTAGT | Simon, 2006 |
|  |  | N-200 | ACCTTTATAARTGGGGTATGARCC | Simon, 2006 |
| Specific primers | 12 | TN-10-J-3312 | TTCAGTAAAAGCCATCGGTC |  |
|  |  | TN-10-N-4851 | TGAAATGTTGCTTCTCGGAT |  |
|  | 13 | TN-10-J-5339 | TAACCACTTTTCCCCCATT |  |
|  |  | TN-10-N-7348 | TTACCTGCTGCTATGGCTG |  |
|  | 14 | TN-10-J-8575 | ATCCCTCTGACGAATACAA |  |
|  |  | TN-10-N-10800 | CATTATCTACTGCGAACCC |  |
|  | 15 | TN-10-J-10717 | CTTTTTGGGGAGCAACAGTA |  |
|  |  | TN-10-N-12768 | TACCTTAGGGATAACAGCGT |  |
|  | 16 | TN-10-J-14457 | GCTTGTATAACCGCAACTG |  |
|  |  | TN-10-N-47 | CGTAGTGAAGGTAGATTACTCT |  |
|  | 17 | TN-10-J-14236 | GGTTCCTCTGAATGGACTAA |  |
|  |  | TN-10-N-400 | AATACCAGAGGCTAAGGCT |  |
|  | 18 | TN-11-J-14208 | ACAGGTTCCTCTGAATAGAC |  |
|  |  | TN-11-N-398 | TCTTGATGCTAATGCTTGAG |  |
|  | 19 | TN-11-J-3415 | AGATGTAGATAACCGCACTG |  |
|  |  | TN-11-N-4529 | TATTGAGAGTGGGACCTGT |  |
|  | 20 | TN-11-J-5384 | GCTGCTGCTTGATATTGAC |  |
|  |  | TN-11-N-7620 | GGATGAGATGGTTTAGGTTTAG |  |
|  | 21 | TN-11-J-8731 | TAGAACCTGATACTGGAGCC |  |
|  |  | TN-11-N-10845 | CTGAAACAATGAAGGGAAAT |  |
|  | 22 | TN-11-J-10822 | CGATTCTTCACATTCCATTT |  |
|  |  | TN-11-N-12861 | TATTTGGTTGGGGTGATTG |  |
|  | 23 | TN-11-J-3188 | GTTTGAACCATTCTCCCTGC |  |
|  |  | TN-11-N-4493 | AGTGGGACCTGTATTTCCTAAG |  |
|  | 24 | TN-11-J-11367 | CTGTAACTGCTAAAACTCAAGA |  |
|  |  | TN-11-N-12999 | GAATGAATGGTTGGATGAA |  |
|  | 25 | TN-11-J-15443 TCTACTCTAAATCCAGCACAAA | TCCACGGTCTTCATGTAATC |  |
|  |  | TN-11-N-384 CTTGATGCTAATGCTTGAGTAA | CTGCTATGGCTGCTCCTA |  |
|  | 26 | TN-5-J-2882 | TTACCTCCTGCTGAACATAG |  |
|  |  | TN-5-N-4578 | GACTGAATGATAGATACGGCT |  |
|  | 27 | TN-5-J-5282 | TCCACGGTCTTCATGTAATC |  |
|  |  | TN-5-N-7347 CTGCTATGGCTGCTCCTA | CTGCTATGGCTGCTCCTA |  |
|  | 28 | TN-5-J-8716 | CTGAAACTGGAGCCTCTACA |  |
|  |  | TN-5-N-10877 | AGAGGATTGTTTGAACCAGT |  |
|  | 29 | TN-5-J-10899 | AACTGGTTCAAACAATCCTC |  |
|  |  | TN-5-N-12942 | TAAAAGACGAGAAGACCCTA |  |
|  | 30 | TN-5-J-14227 | GACTAAAAAACCGCCAAAA |  |
|  |  | TN-5-N-531 | GGAATCAAGTATGAAATGGG |  |
|  | 31 | TN-5-J-11306 | CGACCAGTTGAAGACCCATA |  |
|  |  | TN-5-N-12737 | ACCTTAGGGATAACAGCGTA |  |
|  | 32 | TN-5-J-2713 | CTGATTATCCCGATGCTTT |  |
|  |  | TN-5-N-4578 | AGACTGAATGATAGATACGGCT |  |
|  | 33 | TN-5-J-2783 | ATTACCTCCTGCTGAACATAG |  |
|  |  | TN-5-N-4579 | AGACTGAATGATAGATACGGC |  |
|  | 34 | TN-9-J-3404 | TTCGTCTTCTTGATGTGGAT |  |
|  |  | TN-9-N-4666 | ACTAAGTGGAAAGGGTGATT |  |
|  | 35 | TN-9-J-8659 | AATCGTATCATTCCGTAACC |  |
|  |  | TN-9-N-10813 | ATGAAGGGAAATAGAAAGTGA |  |
|  | 36 | TN-9-J-5383 GCAGCCTGATACTGACATT | TTCAGTAAAAGCCATCGGTC |  |
|  |  | TN-9-N-6652 TGGTGGTTAGTGGGAGTAT | TGAAATGTTGCTTCTCGGAT |  |
|  | 37 | TN-9-J-10781 TGAGGAGGATTTGCTGTTG | TAACCACTTTTCCCCCATT |  |
|  |  | TN-9-N-12694 CGAAAAAAGAGTTTGCGAC | TTACCTGCTGCTATGGCTG |  |
|  | 38 | TN-9-J-14177 ATCGTGGACTATCATTTACAG | ATCCCTCTGACGAATACAA |  |
|  |  | TN-9-N-324 TTCTATCTCTCAATAAGGGGA | CATTATCTACTGCGAACCC |  |
|  | 39 | TN-9-J-5358 GCWGCWGCTTGATATTGAC | CTTTTTGGGGAGCAACAGTA |  |
|  |  | TN-9-N-7604 GGWTGAGATGGWTTAGGWTTRG | TACCTTAGGGATAACAGCGT |  |
|  | 40 | TN-9-J-8657 TAATCGTATCATTCCGTAACC | GTTTGAACCATTCTCCCTGC |  |
|  |  | TN-9-N-9424 TAAGGGTTTGTGAGGGAGTT | AGTGGGACCTGTATTTCCTAAG |  |
|  | 40 | TN-3-J-3399 | ACTAATGAACTAAAGCCCCA |  |
|  |  | TN-3-N-4676 | GGTGATTTTTGTGTGAGTGC |  |
|  | 41 | TN-3-J-5364 | ATTCACCACTTTGGATTCG |  |
|  |  | TN-3-N-6607 | TTTTAGTGTCTGGCGTTGG |  |
|  | 42 | TN-3-J-8783 | GAGCCTTAGGTAATCACAGA |  |
|  |  | TN-3-N-11087 | TGTTACAAGTGGGTTAGCAG |  |
|  | 43 | TN-3-J-10936 | CCCTTAGGAGTAAACGGAAA |  |
|  |  | TN-3-N-12968 | AAAAGACGAGAAGACCCTAT |  |
|  | 44 | TN-3-J-13937 | ACGCCTACTATGTTACGACT |  |
|  |  | TN-3-N-702 | CGTAGACTTACTTGGTTTAGG |  |
|  | 45 | TN-3-J-5384 | GCTGCTGCTTGATATTGAC |  |
|  |  | TN-3-N-7620 | GGATGAGATGGTTTAGGTTTAG |  |

Note: The primers starting with TN-3, TN-5, TN-9, TN-10, TN-11 are specific primers of *Oberea yaoshana,* *Thermistis croccocincta,* *Blepephaeus succinctor,* *Nortia carinicollis,* *Pterolophia* sp.ZJY-2019, respectively.

Except for TN5-J-5282/TN5-N-7347 used for *Oberea yaoshana* and *Thermistis croccocincta,* TN-3-J-5384/TN-3-N-7620 used for *Oberea yaoshana*, *Thermistis croccocincta* and *Blepephaeus succinctor.*
