# Supplementary material for: The complete mitochondrial genomes of five longicorn beetles (Coleoptera: Cerambycidae) and phylogenetic relationships within Cerambycidae
Source: PeerJ. 2019 Sep 5;7:e7633. doi: 10.7717/peerj.7633 (PMC6732212; doi:10.7717/peerj.7633)
Supplement: Supplemental Information 3 [file peerj-07-7633-s009.docx]

| Gene | Strand | Position | Length  (nuc.) | Anti  Codon | Start  Codon | Stop  Codon | Intergenic  nucleotides |
| --- | --- | --- | --- | --- | --- | --- | --- |
| tRNA^Ile^ | + | 1-65 | 65 | ATC |  |  | 0 |
| tRNA^Gln^ | - | 63-131 | 69 | CAA |  |  | -3 |
| tRNA^Met^ | + | 132-200 | 69 | ATG |  |  | 0 |
| *nad2* | + | 201-1211 | 1011 |  | ATT | TAA | 0 |
| tRNA^Trp^ | + | 1210-1273 | 64 | TGA |  |  | -2 |
| tRNA^Cys^ | - | 1266-1327 | 62 | TGC |  |  | -8 |
| tRNA^Tyr^ | - | 1333-1397 | 65 | TAC |  |  | +5 |
| *cox1* | + | 1393-2932 | 1540 |  | ATA | T | -5 |
| tRNA^Leu2^ | + | 2933-2997 | 65 | TAA |  |  | 0 |
| *cox2* | + | 2998-3685 | 688 |  | ATT | T | 0 |
| tRNA^Lys^ | + | 3686-3754 | 69 | AAA |  |  | 0 |
| tRNA^Asp^ | + | 3755-3820 | 66 | GAC |  |  | 0 |
| *atp8* | + | 3821-3976 | 156 |  | ATT | TAA | 0 |
| *atp6* | + | 3973-4644 | 672 |  | ATA | TAA | -4 |
| *cox3* | + | 4644-5432 | 789 |  | ATG | TAA | -1 |
| tRNA^Gly^ | + | 5435-5498 | 64 | GCA |  |  | +2 |
| *nad3* | + | 5499-5852 | 354 |  | ATT | TAG | 0 |
| tRNA^Ala^ | + | 5851-5917 | 67 | GCA |  |  | -2 |
| tRNA^Arg^ | + | 5918-5982 | 65 | CGA |  |  | 0 |
| tRNA^Asn^ | + | 5982-6047 | 66 | AAC |  |  | -1 |
| tRNA^Ser1^ | + | 6048-6114 | 67 | AGA |  |  | 0 |
| tRNA^Glu^ | + | 6115-6176 | 62 | GAA |  |  | 0 |
| tRNA^Phe^ | - | 6175-6239 | 65 | TTC |  |  | -2 |
| *nad5* | - | 6240-7953 | 1714 |  | ATT | T | 0 |
| tRNA^His^ | - | 7954-8017 | 64 | CAC |  |  | 0 |
| *nad4* | - | 8018-9350 | 1333 |  | ATG | T | 0 |
| *nad4l* | - | 9349-9658 | 310 |  | ATT | T | -2 |
| tRNA^Thr^ | + | 9659-9722 | 64 | ACA |  |  | 0 |
| tRNA^Pro^ | - | 9723-9789 | 67 | CCA |  |  | 0 |
| *nad6* | + | 9792-10292 | 501 |  | ATC | TAA | +2 |
| *cytb* | + | 10291-11431 | 1140 |  | ATG | TAA | -2 |
| tRNA^Ser2^ | + | 11432-11499 | 68 | TCA |  |  | 0 |
| *nad1* | - | 11517-12467 | 951 |  | TTG | TAG | +17 |
| tRNA^Leu1^ | - | 12469-12532 | 64 | CTA |  |  | +1 |
| 16S rRNA | - | 12533-13815 | 1283 |  |  |  | 0 |
| tRNA^Val^ | - | 13816-13884 | 69 | GTA |  |  | 0 |
| 12S rRNA | - | 13880-14648 | 769 |  |  |  | +1 |
| CR | + | 14649-15529 | 861 |  |  |  | 0 |
