# Supplementary material for: The complete mitochondrial genomes of five longicorn beetles (Coleoptera: Cerambycidae) and phylogenetic relationships within Cerambycidae
Source: PeerJ. 2019 Sep 5;7:e7633. doi: 10.7717/peerj.7633 (PMC6732212; doi:10.7717/peerj.7633)
Supplement: Supplemental Information 4 [file peerj-07-7633-s010.docx]

| Gene | Strand | Position | Length  (nuc.) | Anti  Codon | Start  Codon | Stop  Codon | Intergenic  nucleotides |
| --- | --- | --- | --- | --- | --- | --- | --- |
| tRNA^Ile^ | + | 1-64 | 64 | ATC |  |  | 0 |
| tRNA^Gln^ | - | 71-139 | 69 | CAA |  |  | +6 |
| tRNA^Met^ | + | 140-208 | 69 | ATG |  |  | 0 |
| *nad2* | + | 209-1219 | 1011 |  | ATT | TAA | 0 |
| tRNA^Trp^ | + | 1218-1284 | 67 | TGA |  |  | -2 |
| tRNA^Cys^ | - | 1277-1339 | 63 | TGC |  |  | -8 |
| tRNA^Tyr^ | - | 1344-1409 | 66 | TAC |  |  | +4 |
| *cox1* | + | 1405-2949 | 1545 |  | ATA | TAA | -5 |
| tRNA^Leu2^ | + | 2945-3009 | 65 | TAA |  |  | -5 |
| *cox2* | + | 3010-3697 | 688 |  | ATC | T | 0 |
| tRNA^Lys^ | + | 3698-3767 | 70 | AAA |  |  | 0 |
| tRNA^Asp^ | + | 3768-3831 | 64 | GAC |  |  | 0 |
| *atp8* | + | 3832-3987 | 156 |  | ATT | TAA | 0 |
| *atp6* | + | 3987-4655 | 669 |  | ATA | TAA | -1 |
| *cox3* | + | 4655-5443 | 789 |  | ATG | TAA | -1 |
| tRNA^Gly^ | + | 5454-5517 | 64 | GCA |  |  | +10 |
| *nad3* | + | 5518-5871 | 354 |  | ATT | TAG | 0 |
| tRNA^Ala^ | + | 5870-5934 | 65 | GCA |  |  | -2 |
| tRNA^Arg^ | + | 5935-5995 | 61 | CGA |  |  | 0 |
| tRNA^Asn^ | + | 5996-6061 | 66 | AAC |  |  | 0 |
| tRNA^Ser1^ | + | 6062-6130 | 69 | AGA |  |  | 0 |
| tRNA^Glu^ | + | 6131-6194 | 64 | GAA |  |  | 0 |
| tRNA^Phe^ | - | 6195-6258 | 64 | TTC |  |  | 0 |
| *nad5* | - | 6259-7972 | 1714 |  | ATT | T | 0 |
| tRNA^His^ | - | 7973-8038 | 66 | CAC |  |  | 0 |
| *nad4* | - | 8039-9368 | 1330 |  | ATG | T | 0 |
| *nad4l* | - | 9367-9649 | 283 |  | ATG | T | -2 |
| tRNA^Thr^ | + | 9652-9715 | 64 | ACA |  |  | +2 |
| tRNA^Pro^ | - | 9716-9779 | 64 | CCA |  |  | 0 |
| *nad6* | + | 9782-10285 | 504 |  | ATT | TAA | +2 |
| *cytb* | + | 10285-11427 | 1143 |  | ATG | TAG | -1 |
| tRNA^Ser2^ | + | 11426-11492 | 67 | TCA |  |  | -2 |
| *nad1* | - | 11510-12460 | 951 |  | TTG | TAG | +17 |
| tRNA^Leu1^ | - | 12462-12526 | 65 | CTA |  |  | +1 |
| 16S rRNA | - | 12527-13807 | 1281 |  |  |  | 0 |
| tRNA^Val^ | - | 13808-13876 | 69 | GTA |  |  | 0 |
| 12S rRNA | - | 13879-14663 | 785 |  |  |  | +2 |
| CR | + | 14664-15554 | 891 |  |  |  | 0 |
