# Supplementary material for: The complete mitochondrial genomes of five longicorn beetles (Coleoptera: Cerambycidae) and phylogenetic relationships within Cerambycidae
Source: PeerJ. 2019 Sep 5;7:e7633. doi: 10.7717/peerj.7633 (PMC6732212; doi:10.7717/peerj.7633)
Supplement: Supplemental Information 5 [file peerj-07-7633-s011.docx]

| Gene | Strand | Position | Length  (nuc.) | Anti  codon | Start  codon | Stop  codon | Intergenic  nucleotides |
| --- | --- | --- | --- | --- | --- | --- | --- |
| tRNA^Ile^ | + | 1-65 | 65 | ATC |  |  | 0 |
| tRNA^Gln^ | - | 63-131 | 69 | CAA |  |  | -3 |
| tRNA^Met^ | + | 131-199 | 69 | ATG |  |  | -1 |
| *nad2* | + | 200-1213 | 1014 |  | ATT | TAA | 0 |
| tRNA^Trp^ | + | 1212-1276 | 65 | TGA |  |  | -2 |
| tRNA^Cys^ | - | 1276-1337 | 62 | TGC |  |  | -1 |
| tRNA^Tyr^ | - | 1338-1403 | 66 | TAC |  |  | 0 |
| *cox1* | + | 1396-2940 | 1545 |  | ATT | TAA | -8 |
| tRNA^Leu2^ | + | 2942-3005 | 64 | TAA |  |  | +1 |
| *cox2* | + | 3006-3686 | 681 |  | ATC | TAA | 0 |
| tRNA^Lys^ | + | 3689-3758 | 70 | AAA |  |  | +2 |
| tRNA^Asp^ | + | 3759-3820 | 62 | GAC |  |  | 0 |
| *atp8* | + | 3821-3976 | 156 |  | ATT | TAG | 0 |
| *atp6* | + | 3973-4641 | 669 |  | ATA | TAA | -4 |
| *cox3* | + | 4645-5431 | 787 |  | ATG | T | +3 |
| tRNA^Gly^ | + | 5432-5495 | 64 | GGA |  |  | 0 |
| *nad3* | + | 5496-5849 | 354 |  | ATT | TAG | -2 |
| tRNA^Ala^ | + | 5848-5911 | 64 | GCA |  |  | 0 |
| tRNA^Arg^ | + | 5911-5973 | 63 | CGA |  |  | -1 |
| tRNA^Asn^ | + | 5973-6036 | 64 | AAC |  |  | -1 |
| tRNA^Ser1^ | + | 6037-6103 | 67 | AGA |  |  | 0 |
| tRNA^Glu^ | + | 6104-6166 | 63 | GAA |  |  | 0 |
| tRNA^Phe^ | - | 6165-6229 | 65 | TTC |  |  | -2 |
| *nad5* | - | 6230-7949 | 1720 |  | ATT | T | 0 |
| tRNA^His^ | - | 7950-8013 | 64 | CAC |  |  | 0 |
| *nad4* | - | 8013-9344 | 1332 |  | ATA | TAA | +1 |
| *nad4l* | - | 9341-9622 | 282 |  | ATG | TAA | -4 |
| tRNA^Thr^ | + | 9626-9688 | 63 | ACA |  |  | +3 |
| tRNA^Pro^ | - | 9689-9752 | 64 | CCA |  |  | 0 |
| *nad6* | + | 9754-10261 | 508 |  | ATA | T | +1 |
| *cytb* | + | 10260-11391 | 1132 |  | ATA | T | -2 |
| tRNA^Ser2^ | + | 11392-11459 | 68 | TCA |  |  | 0 |
| *nad1* | - | 11477-12424 | 948 |  | TTG | TAG | +17 |
| tRNA^Leu1^ | - | 12426-12490 | 65 | CTA |  |  | +1 |
| 16S rRNA | - | 12491-13751 | 1261 |  |  |  | 0 |
| tRNA^Val^ | - | 13752-13820 | 69 | GTA |  |  | 0 |
| 12S rRNA | - | 13823-14594 | 772 |  |  |  | +2 |
| CR | + | 14595-15602 | 1008 |  |  |  | 0 |
