# Supplementary material for: The complete mitochondrial genomes of five longicorn beetles (Coleoptera: Cerambycidae) and phylogenetic relationships within Cerambycidae
Source: PeerJ. 2019 Sep 5;7:e7633. doi: 10.7717/peerj.7633 (PMC6732212; doi:10.7717/peerj.7633)
Supplement: Supplemental Information 6 [file peerj-07-7633-s012.docx]

| Gene | Strand | Position | | | Length  (nuc.) | Anti  Codon | Start  Codon | Stop  Codon | Intergenic  nucleotides |
| --- | --- | --- | --- | --- | --- | --- | --- | --- | --- |
| tRNA^Ile^ | + | 1 | 65 | | 65 | ATC |  |  | 0 |
| tRNA^Gln^ | - | 67 | 135 | | 69 | CAA |  |  | +1 |
| tRNA^Met^ | + | 135 | 203 | | 69 | ATG |  |  | -1 |
| *nad2* | + | 204 | 1211 | | 1008 |  | ATT | TAA | -2 |
| tRNA^Trp^ | + | 1210 | 1282 | | 73 | TGA |  |  | 0 |
| tRNA^Cys^ | - | 1275 | 1336 | | 62 | TGC |  |  | -8 |
| tRNA^Tyr^ | - | 1521 | 1586 | | 66 | TAC |  |  | +184 |
| *cox1* | + | 1579 | 3121 | | 1543 |  | TGG | T | -8 |
| tRNA^Leu2^ | + | 3122 | 3185 | | 64 | TAA |  |  | 0 |
| *cox2* | + | 3186 | 3870 | | 685 |  | ATT | T | 0 |
| tRNA^Lys^ | + | 3871 | 3940 | | 70 | AAA |  |  | -1 |
| tRNA^Asp^ | + | 3941 | 4004 | | 64 | GAC |  |  | 0 |
| *atp8* | + | 4005 | 4160 | | 156 |  | ATT | TAA | +1 |
| *atp6* | + | 4157 | 4828 | | 672 |  | ATA | TAA | -4 |
| *cox3* | + | 4828 | 5614 | | 787 |  | ATG | T | -1 |
| tRNA^Gly^ | + | 5615 | 5681 | | 67 | GCA |  |  | -1 |
| *nad3* | + | 5682 | 6035 | | 354 |  | ATA | TAG | -2 |
| tRNA^Ala^ | + | 6034 | 6101 | | 68 | GCA |  |  | -1 |
| tRNA^Arg^ | + | 6102 | 6165 | | 64 | CGA |  |  | 0 |
| tRNA^Asn^ | + | 6166 | 6230 | | 65 | AAC |  |  | 0 |
| tRNA^Ser1^ | + | 6231 | 6296 | | 66 | AGA |  |  | 0 |
| tRNA^Glu^ | + | 6297 | 6359 | | 63 | GAA |  |  | 0 |
| tRNA^Phe^ | - | 6360 | 6424 | | 65 | TTC |  |  | 0 |
| *nad5* | - | 6425 | 8141 | | 1717 |  | ATT | T | +1 |
| tRNA^His^ | - | 8142 | 8205 | | 64 | CAC |  |  | -1 |
| *nad4* | - | 8205 | 9536 | | 1332 |  | ATG | TAA | 0 |
| *nad4l* | - | 9530 | 9817 | | 288 |  | ATG | TAA | -7 |
| tRNA^Thr^ | + | 9820 | 9883 | | 64 | ACA |  |  | +1 |
| tRNA^Pro^ | - | 9884 | 9950 | | 67 | CCA |  |  | 0 |
| *nad6* | + | 9953 | 10456 | | 504 |  | ATT | TAA | +3 |
| *cytb* | + | 10456 | 11598 | | 1143 |  | ATG | TAA | -1 |
| tRNA^Ser2^ | + | 11598 | 11668 | | 71 | TCA |  |  | -2 |
| *nad1* | - | 11826 | 12767 | | 942 |  | TTG | TAA | +157 |
| tRNA^Leu1^ | - | 12769 | 12834 | | 66 | CTA |  |  | 0 |
| 16S rRNA | - | 12835 | 14116 | | 1282 |  |  |  | 0 |
| tRNA^Val^ | - | 14117 | 14185 | | 69 | GTA |  |  | 0 |
| 12S rRNA | - | 14189 | 14926 | | 738 |  |  |  | +4 |
| CR | + | 14927 | | 16063 | 1137 |  |  |  |  |
