# Supplementary material for: The complete mitochondrial genomes of five longicorn beetles (Coleoptera: Cerambycidae) and phylogenetic relationships within Cerambycidae
Source: PeerJ. 2019 Sep 5;7:e7633. doi: 10.7717/peerj.7633 (PMC6732212; doi:10.7717/peerj.7633)
Supplement: Supplemental Information 7 [file peerj-07-7633-s013.docx]

| Codon(AA) | *O. yaoshana* | | *T. croccocincta* | | *B. succinctor* | | *N. carinicollis* | | *Pterolophia* sp.ZJY-2019 | |
| --- | --- | --- | --- | --- | --- | --- | --- | --- | --- | --- |
|  | Count | RSCU | Count | RSCU | Count | RSCU | Count | RSCU | Count | RSCU |
| UUU(F) | 313 | 1.86 | 300 | 1.81 | 279 | 1.72 | 309 | 1.72 | 282 | 1.77 |
| UUC(F) | 23 | 0.14 | 31 | 0.19 | 46 | 0.28 | 50 | 0.28 | 36 | 0.23 |
| UUA(L) | 463 | 4.97 | 426 | 4.53 | 346 | 3.77 | 290 | 3.2 | 401 | 4.2 |
| UUG(L) | 13 | 0.14 | 24 | 0.26 | 47 | 0.51 | 53 | 0.58 | 30 | 0.31 |
| CUU(L) | 45 | 0.48 | 68 | 0.72 | 79 | 0.86 | 92 | 1.01 | 85 | 0.89 |
| CUC(L) | 2 | 0.02 | 8 | 0.09 | 11 | 0.12 | 27 | 0.3 | 14 | 0.15 |
| CUA(L) | 36 | 0.39 | 38 | 0.4 | 63 | 0.69 | 72 | 0.79 | 41 | 0.43 |
| CUG(L) | 0 | 0 | 0 | 0 | 4 | 0.04 | 10 | 0.11 | 2 | 0.02 |
| AUU(I) | 384 | 1.9 | 364 | 1.82 | 328 | 1.76 | 287 | 1.76 | 365 | 1.85 |
| AUC(I) | 21 | 0.1 | 35 | 0.18 | 45 | 0.24 | 40 | 0.24 | 29 | 0.15 |
| AUA(M) | 205 | 1.92 | 177 | 1.79 | 173 | 1.75 | 168 | 1.72 | 170 | 1.82 |
| AUG(M) | 9 | 0.08 | 21 | 0.21 | 25 | 0.25 | 27 | 0.28 | 17 | 0.18 |
| GUU(V) | 71 | 1.88 | 87 | 2.27 | 94 | 2.03 | 90 | 1.76 | 102 | 2.31 |
| GUC(V) | 1 | 0.03 | 8 | 0.21 | 7 | 0.15 | 15 | 0.29 | 5 | 0.11 |
| GUA(V) | 75 | 1.99 | 56 | 1.46 | 68 | 1.47 | 91 | 1.78 | 66 | 1.49 |
| GUG(V) | 4 | 0.11 | 2 | 0.05 | 16 | 0.35 | 9 | 0.18 | 4 | 0.09 |
| UAU(Y) | 146 | 1.91 | 147 | 1.88 | 127 | 1.62 | 128 | 1.67 | 136 | 1.83 |
| UAC(Y) | 7 | 0.09 | 9 | 0.12 | 30 | 0.38 | 25 | 0.33 | 13 | 0.17 |
| CAU(H) | 65 | 1.91 | 65 | 1.86 | 64 | 1.71 | 43 | 1.23 | 64 | 1.73 |
| CAC(H) | 3 | 0.09 | 5 | 0.14 | 11 | 0.29 | 27 | 0.77 | 10 | 0.27 |
| CAA(Q) | 63 | 1.94 | 65 | 1.86 | 63 | 1.85 | 50 | 1.49 | 53 | 1.83 |
| CAG(Q) | 2 | 0.06 | 5 | 0.14 | 5 | 0.15 | 17 | 0.51 | 5 | 0.17 |
| AAU(N) | 182 | 1.92 | 166 | 1.87 | 137 | 1.68 | 126 | 1.58 | 158 | 1.83 |
| AAC(N) | 8 | 0.08 | 12 | 0.13 | 26 | 0.32 | 33 | 0.42 | 15 | 0.17 |
| AAA(K) | 91 | 1.84 | 89 | 1.85 | 85 | 1.68 | 71 | 1.61 | 84 | 1.75 |
| AAG(K) | 8 | 0.16 | 7 | 0.15 | 16 | 0.32 | 17 | 0.39 | 12 | 0.25 |
| GAU(D) | 55 | 1.8 | 58 | 1.76 | 59 | 1.79 | 49 | 1.53 | 56 | 1.78 |
| GAC(D) | 6 | 0.2 | 8 | 0.24 | 7 | 0.21 | 15 | 0.47 | 7 | 0.22 |
| GAA(E) | 70 | 1.87 | 66 | 1.86 | 69 | 1.77 | 66 | 1.59 | 65 | 1.67 |
| GAG(E) | 5 | 0.13 | 5 | 0.14 | 9 | 0.23 | 17 | 0.41 | 13 | 0.33 |
| UCU(S) | 82 | 2 | 92 | 2.19 | 111 | 2.76 | 109 | 2.49 | 122 | 3.03 |
| UCC(S) | 15 | 0.37 | 10 | 0.24 | 20 | 0.5 | 27 | 0.62 | 13 | 0.32 |
| UCA(S) | 93 | 2.27 | 93 | 2.21 | 55 | 1.37 | 89 | 2.03 | 72 | 1.79 |
| UCG(S) | 3 | 0.07 | 4 | 0.1 | 4 | 0.1 | 11 | 0.25 | 0 | 0 |
| CCU(P) | 67 | 2.21 | 67 | 2.18 | 69 | 2.32 | 47 | 1.49 | 66 | 2.24 |
| CCC(P) | 18 | 0.6 | 19 | 0.62 | 24 | 0.81 | 38 | 1.21 | 10 | 0.34 |
| CCA(P) | 36 | 1.19 | 35 | 1.14 | 23 | 0.77 | 36 | 1.14 | 42 | 1.42 |
| CCG(P) | 0 | 0 | 2 | 0.07 | 3 | 0.1 | 5 | 0.16 | 0 | 0 |
| ACU(T) | 88 | 2.13 | 65 | 1.58 | 93 | 2.13 | 61 | 1.53 | 90 | 2.09 |
| ACC(T) | 10 | 0.24 | 21 | 0.51 | 26 | 0.59 | 28 | 0.7 | 20 | 0.47 |
| ACA(T) | 66 | 1.6 | 76 | 1.84 | 52 | 1.19 | 69 | 1.74 | 62 | 1.44 |
| ACG(T) | 1 | 0.02 | 3 | 0.07 | 4 | 0.09 | 1 | 0.03 | 0 | 0 |
| GCU(A) | 76 | 2.22 | 77 | 2.25 | 95 | 2.36 | 53 | 1.42 | 91 | 2.32 |
| GCC(A) | 8 | 0.23 | 14 | 0.41 | 27 | 0.67 | 21 | 0.56 | 17 | 0.43 |
| GCA(A) | 51 | 1.49 | 45 | 1.31 | 35 | 0.87 | 71 | 1.91 | 48 | 1.22 |
| GCG(A) | 2 | 0.06 | 1 | 0.03 | 4 | 0.1 | 4 | 0.11 | 1 | 0.03 |
| UGU(C) | 27 | 1.74 | 27 | 1.74 | 27 | 1.8 | 26 | 1.58 | 26 | 1.86 |
| UGC(C) | 4 | 0.26 | 4 | 0.26 | 3 | 0.2 | 7 | 0.42 | 2 | 0.14 |
| UGA(W) | 79 | 1.88 | 86 | 1.91 | 78 | 1.84 | 68 | 1.6 | 80 | 1.86 |
| UGG(W) | 5 | 0.12 | 4 | 0.09 | 7 | 0.16 | 17 | 0.4 | 6 | 0.14 |
| CGU(R) | 24 | 1.81 | 16 | 1.23 | 22 | 1.69 | 14 | 1.04 | 22 | 1.63 |
| CGC(R) | 0 | 0 | 0 | 0 | 2 | 0.15 | 3 | 0.22 | 1 | 0.07 |
| CGA(R) | 27 | 2.04 | 35 | 2.69 | 24 | 1.85 | 31 | 2.3 | 28 | 2.07 |
| CGG(R) | 2 | 0.15 | 1 | 0.08 | 4 | 0.31 | 6 | 0.44 | 3 | 0.22 |
| AGU(S) | 31 | 0.76 | 34 | 0.81 | 26 | 0.65 | 26 | 0.59 | 28 | 0.7 |
| AGC(S) | 2 | 0.05 | 1 | 0.02 | 3 | 0.07 | 7 | 0.16 | 3 | 0.07 |
| AGA(S) | 96 | 2.34 | 97 | 2.31 | 84 | 2.09 | 63 | 1.44 | 70 | 1.74 |
| AGG(S) | 6 | 0.15 | 5 | 0.12 | 19 | 0.47 | 18 | 0.41 | 14 | 0.35 |
| GGU(G) | 44 | 0.95 | 57 | 1.19 | 58 | 1.18 | 52 | 0.99 | 50 | 0.99 |
| GGC(G) | 8 | 0.17 | 8 | 0.17 | 22 | 0.45 | 13 | 0.25 | 9 | 0.18 |
| GGA(G) | 117 | 2.53 | 117 | 2.45 | 99 | 2.01 | 106 | 2.02 | 117 | 2.31 |
| GGG(G) | 16 | 0.35 | 9 | 0.19 | 18 | 0.37 | 39 | 0.74 | 27 | 0.53 |
